# A Comparison for Dimensionality Reduction Methods of Single-Cell RNA-seq Data

**DOI:** 10.3389/fgene.2021.646936

**Published:** 2021-03-23

**Authors:** Ruizhi Xiang, Wencan Wang, Lei Yang, Shiyuan Wang, Chaohan Xu, Xiaowen Chen

**Affiliations:** ^1^College of Bioinformatics Science and Technology, Harbin Medical University, Harbin, China; ^2^School of Optometry and Ophthalmology and Eye Hospital, Wenzhou Medical University, Wenzhou, China

**Keywords:** single-cell RNA-seq, dimension reduction, benchmark, sequences analysis, deep learning

## Abstract

Single-cell RNA sequencing (scRNA-seq) is a high-throughput sequencing technology performed at the level of an individual cell, which can have a potential to understand cellular heterogeneity. However, scRNA-seq data are high-dimensional, noisy, and sparse data. Dimension reduction is an important step in downstream analysis of scRNA-seq. Therefore, several dimension reduction methods have been developed. We developed a strategy to evaluate the stability, accuracy, and computing cost of 10 dimensionality reduction methods using 30 simulation datasets and five real datasets. Additionally, we investigated the sensitivity of all the methods to hyperparameter tuning and gave users appropriate suggestions. We found that t-distributed stochastic neighbor embedding (t-SNE) yielded the best overall performance with the highest accuracy and computing cost. Meanwhile, uniform manifold approximation and projection (UMAP) exhibited the highest stability, as well as moderate accuracy and the second highest computing cost. UMAP well preserves the original cohesion and separation of cell populations. In addition, it is worth noting that users need to set the hyperparameters according to the specific situation before using the dimensionality reduction methods based on non-linear model and neural network.

## Introduction

The technological advances in single-cell RNA sequencing (scRNA-seq) have allowed to measure the DNA and/or RNA molecules in single cells, enabling us to identify novel cell types, cell states, trace development lineages, and reconstruct the spatial organization of cells (Hedlund and Deng, 2018). Single-cell technology has become a research hotspot. However, such analysis heavily relies on the accurate similarity assessment of a pair of cells, which poses unique challenges such as outlier cell populations, transcript amplification noise, and dropout events. Additionally, single-cell datasets are typically high dimensional in large numbers of measured cells. For example, scRNA-seq can theoretically measure the expression of all the genes in tens of thousands of cells in a single experiment (Wagner et al., 2016). Although whole-transcriptome analyses avoid the bias of using a predefined gene set (Jiang et al., 2015), the dimensionality of such datasets is typically too high for most modeling algorithms to process directly. Moreover, biological systems own the lower intrinsic dimensionality. For example, a differentiating hematopoietic cell can be represented by two or more dimensions: one denotes how far it has progressed in its differentiation toward a particular cell type, and at least another dimension denotes its current cell-cycle stage. Therefore, dimensionality reduction is necessary to project high-dimensional data into low-dimensional space to visualize the cluster structures and development trajectory inference.

Research on data dimension reduction has a long history, and principal component analysis (PCA), which is still widely used, can be traced back to 1901. Since the advent of RNA-seq technology, this linear dimension-reduction method has been favored by researchers. In addition, there are non-linear methods such as uniform manifold approximation and projection (UMAP) and t-distributed stochastic neighbor embedding (t-SNE) to reduce dimension. After the rise of neural network, there are many methods of dimensionality reduction based on neural network such as variational autoencoder (VAE). In addition, there are some new theoretical frameworks such as the multikernel learning [single-cell interpretation *via* multikernel learning (SIMLR)] based on the above methods that have been or are being developed to handle increasingly diverse scRNA-seq data.

**FIGURE 1:**
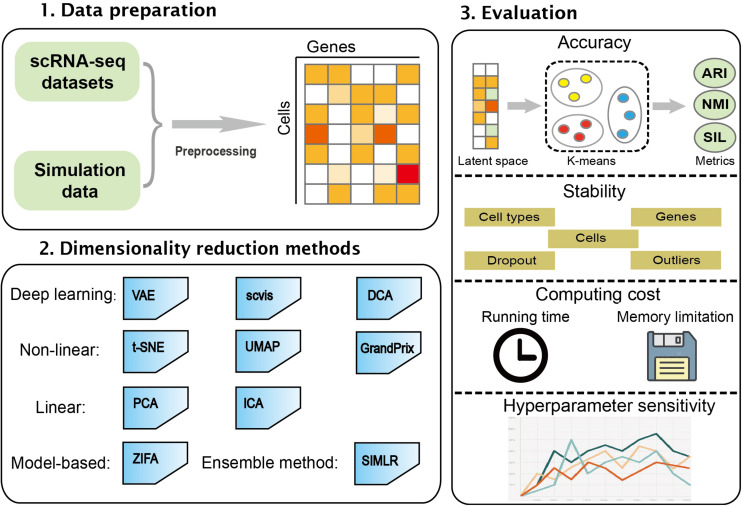
An overview for benchmarking dimensionality reduction methods. The 10 dimensionality reduction methods were evaluated on real scRNA-seq expression datasets and simulation data. k-means was used to cluster low-dimensional latent space. The accuracy, stability, computing cost, and sensitivity to hyperparameters were used to systematically evaluate these methods.

In this study, we performed a comprehensive evaluation of 10 different dimensionality reduction algorithms comprising the linear method, the non-linear method, the neural network, model-based method, and ensemble method. These algorithms were run and compared on simulated and real datasets. The performance of the algorithms was evaluated based on accuracy, stability, computing cost, and sensitivity to hyperparameters. This work will be helpful in developing new algorithms in the field. The workflow of the benchmark framework is shown in Figure 1.

## Materials and Methods

### Methods for Dimensionality Reduction

To our knowledge, about 10 methods are now available to obtain a low-dimensional representation for scRNA-seq data. In this section, we gave an overview of these 10 methods (Table 1).

**TABLE 1:** Summary of dimensionality reduction methods.

**Methods**	**Year**	**Method strategy**	**Platform**	**Input**	**Available URL**	**Version**	**References**
PCA	1987	Linear	R	Counts	R Package Seurat	3.1.0	Jolliffe, 2002
ICA	2001	Linear	R	Counts	R Package Seurat	3.1.0	Liebermeister, 2002
ZIFA	2015	Model-based	Python	Counts	https://github.com/epierson9/ZIFA	0.1	Pierson and Yau, 2015
GrandPrix	2017	Non-linear	Python	1,000 highly genes	https://github.com/ManchesterBioinference/GrandPrix	0.1	Ahmed et al., 2019
t-SNE	2008	Non-linear	R	Counts	R Package Rtsne	0.15	Maaten and Hinton, 2008
UMAP	2018	Non-linear	R/Python	Counts	https://github.com/lmcinnes/umap	0.3.1	McInnes et al., 2018
DCA	2019	Neural network	Python	1,000 Highly genes	https://github.com/theislab/dca	0.2.2	Eraslan et al., 2019
scvis	2018	Neural network	Python	PCA-100	https://bitbucket.org/jerry00/scvis-dev	0.1.0	Ding et al., 2018
VAE	2019	Neural network	Python	Counts	https://github.com/greenelab/CZI-Latent-Assessment/tree/master/single_cell_analysis	NA	Hu and Greene, 2019
SIMLR	2017	Ensemble method	R	Counts	https://github.com/BatzoglouLabSU/SIMLR	1.6.0	Wang et al., 2017

#### PCA

As the most widely used dimensionality reduction algorithm, PCA (Jolliffe, 2002) identifies dominant patterns and the linear combinations of the original variables with maximum variance. The basic idea of PCA is to find the first principal component with the largest variance in the data and then seek the second component in the same way, which is uncorrelated with the first component and accounts for the next largest variance. This process repeats until the new component is almost ineffective or reaches the threshold set by users.

#### ICA

Independent component analysis (ICA) (Liebermeister, 2002), also known as blind source separation (BSS), is a statistical calculation technique used to reveal the factors behind random variables, measured values, and signals. ICA linearly transforms the variables (corresponding to the cells) into independent components with minimal statistical dependencies between them. Unlike PCA, ICA requires the source signal to meet the following two conditions: (1) source signals are independent of each other and (2) the values in each source signal have a non-Gaussian distribution. It assumes that the observed stochastic signal *x* obeys the model *x* = *As*, where *s* is the unknown source signal, its components are independent of each other, and *A* is an unknown mixing matrix. The purpose of the ICA is to estimate the mixing matrix *A* and the source signal *s* by and only by observing *x*.

#### ZIFA

The dropout events in scRNA-seq data may make the classic dimensionality reduction algorithm unsuitable. Pierson and Yau (2015) modified the factor analysis framework to solve the dropout problem and provided a method zero-inflated factor analysis (ZIFA) based on an additional zero-inflation modulation layer for reducing the dimension of single-cell gene expression data. Compared with the above two linear methods, employing the zero-inflation model can give ZIFA more powerful projection capabilities but will pay a corresponding cost in computational complexity.

In the statistical model, the expression level of the *j*th gene in the *i*th sample *y*_*ij*_ (*i* = 1,…, *N* and *j* = 1,…,*D*) is described:

$$z_{i}∼\text{Normal}\,\left(0,I\right),$$

$$x_{i}|z_{i}∼\text{Normal}\,\left(A\,z_{i}+\mu ,W\right),$$

$$h_{i\,j}|x_{i\,j}∼\text{Bernoulli}\,\left(p_{0}\right),$$

$$y_{i\,j}=\{\frac{x_{i\,j},\text{if}\,h_{i\,j}=0}{0,\text{if}\,h_{i\,j}=1}$$

where *z_i* is a *K* × 1 data point in a latent low-dimensional space. *A* denotes a *D* × *K* factor loadings matrix, *H* is a *D* × *N* masking matrix, $W=\text{diag}\,\left(\sigma_{1}^{2},\cdots ,\sigma_{D}^{2}\right)$ a *D* × *D* diagonal matrix, and μ is a *D* × 1 mean vector. Dropout probability *p_0* is a function of the latent expression level, $p_{0}=\text{exp}\,?\,\left(-\lambda \,x_{i\,j}^{2}\right)$, where λ is the exponential decay parameter in the zero-inflation model.

Zero-inflated factor analysis adopted the expectation–maximization (EM) algorithm to infer model parameters Θ=(*A*,σ^2^,μ,λ) that maximize the likelihood *p*(*Y*|θ).

#### GrandPrix

GrandPrix (Ahmed et al., 2019) is based on the variational sparse approximation of the Bayesian Gaussian process latent variable model (Titsias and Lawrence, 2010) to project data to lower dimensional spaces. It requires only a small number of inducing points to efficiently generate a full posterior distribution. GrandPrix optimizes the coordinate position in the latent space by maximizing the joint density of the observation data, and then establishes a mapping from low-dimensional space to high-dimensional space.

The expression profile of each gene *y* is modeled as *y_g* is considered a non-linear function of pseudotime which accompanies with some noise ∈:

$$y_{g}=f_{g}\,\left(t,x\right)+\in$$

where

$$f_{g}\,\left(t,x\right)∼G\,P\,\left(0,\sigma^{2}\,k\,\left(\left(t,x\right),{\left(t,x\right)}^{*}\right)\right)$$

$\in ∼N\left(0,\sigma_{\text{noise}}^{2}\right)$ is a Gaussian distribution with variance $\sigma_{\text{noise}}^{2}$, *x* is the extra latent dimension, σ^2^ is the process variance, and *k*(*t*,*t*^∗^) is the covariance function between two distinct pseudotime points *t* and *t*^∗^. GrandPrix employed the variational free energy (VFE) approximation for inference.

#### t-SNE

t-Distributed stochastic neighbor embedding is a state-of-the-art dimensionality reduction algorithm for non-linear data representation that produces a low-dimensional distribution of high-dimensional data (Maaten and Hinton, 2008; Van Der Maaten, 2014). It excels at revealing local structure in high-dimensional data. t-SNE is based on the SNE (Hinton and Roweis, 2002), which starts from converting the high-dimensional Euclidean distances between data points into conditional probabilities that represent similarities. The main idea and the modifications of t-SNE are (1) the symmetric version of SNE and (2) using a Student’s *t* distribution to compute the similarity between two points in the low-dimensional space.

#### UMAP

Uniform manifold approximation and projection is a dimension reduction technique that can be used not only for visualization similarly to t-SNE but also for general non-linear dimension reduction. Compared with t-SNE, UMAP retains more global structure with superior run-time performance (McInnes et al., 2018; Becht et al., 2019).

The algorithm is based on three assumptions about the data: (a) the data are uniformly distributed on the Riemannian manifold; (b) the Riemannian metric is locally constant (or can be approximated); and (c) the manifold is locally connected. According to these assumptions, the manifold with fuzzy topology can be modeled. The embedding is found by searching the low-dimensional projection of the data with the closest equivalent fuzzy topology. In terms of model construction, UMAP includes two steps: (1) building a particular weighted k-neighbor graph using the nearest-neighbor descent algorithm (Dong et al., 2011) and (2) computing a low-dimensional representation which can preserve desired characteristics of this graph.

#### DCA

Deep count autoencoder (DCA) can denoise scRNA-seq data by deep learning (Eraslan et al., 2019). It extends the typical autoencoder approach to solve denoising and imputation tasks in in one step. The autoencoder framework of DCA is composed by default of three hidden layers with neurons of 64, 32, and 64, respectively, with zero-inflated negative binomial (ZINB) loss functions (Salehi and Roudbari, 2015), learning three parameters of the negative binomial distribution: mean, dispersion, and dropout. The inferred mean parameter of the distribution represents the denoised reconstruction and the main output of DCA. The deep leaning framework enables DCA to capture the complexity and non-linearity in scRNA-seq data. Additionally, DCA can be applied to datasets with more than millions of cells. DCA is parallelizable through a graphics processing unit (GPU) to increase the speed.

#### Scvis

Scvis is a statistical model to capture the low-dimensional structures in scRNA-seq (Ding et al., 2018). The assumption of scvis is a high-dimensional gene expression vector *x_n* of cell *n* which can be generated by drawing a sample from the distribution *p*(*x*|*z*,θ). Here, *z* is a low-dimensional latent vector which follows a simple distribution, e.g., a two-dimensional standard normal distribution. The data-point-specific parameters θ are the output of a feedforward neural network. To better visualize the manifold structure of an scRNA-seq dataset, scvis applies t-SNE objective function on the latent *z* distribution as a constraint to make cells with similar expression profiles to be close in the latent space. In addition, scvis also provides log likelihood ratio to measure the quality of embedding, which can potentially be used for outlier detection.

#### VAE

Variational autoencoder is a data-driven, unsupervised model for dimension reduction using an autoencoding framework, built in Keras with a TensorFlow backend (Hu and Greene, 2019). Comparing with a traditional autoencoder, VAE determined non-linear explanatory features over samples through learning two different latent representations: a mean and standard deviation vector encoding.

The model is mainly composed of two connected neural networks, encoder and decoder. The scRNA-seq data are compressed by the encoder and reconstructed by the decoder. The variable probability *Q*(*z*|*X*) is used to approximate the posterior distribution *P*(*z*|*X*), and it is optimized to minimize the Kullback–Leibler divergence between *Q*(*z*|*X*) and *P*(*z*|*X*) and reconstruction loss. Here, the encoder network is designed as a zero- to two-layer fully connected neural network to generate the mean and variance of a Gaussian distribution *q*_θ_(*z*|*X*), and then the representative latent space *z* is sampled from this distribution. The decoder is also a zero- to two-layer fully connected neural network to reconstruct the count matrix.

#### SIMLR

Single-cell interpretation *via* multikernel learning performs dimension reduction through learning a symmetric matrix *S*_*N × N*_ that captures the cell-to-cell similarity from the input scRNA-seq data (Wang et al., 2017). The assumption of SIMLR is that *S*_*N = N*_ should have an approximate block-diagonal structure with *C* blocks if the input cells have *C* cell types. SIMLR learns proper weights for multiple kernels, which are different measures of cell-to-cell distances, and constructs a symmetric similarity matrix.

Specifically, developers first define the distance between cell *i* and cell *j* as*D*(*c*_*i*_,*c*_*j*_):

$$D\,\left(c_{i},c_{j}\right)=2-2\,\sum_{l}w_{l}\,K_{l}\,\left(c_{i},c_{j}\right),\sum_{l}w_{l}=1,w_{l}\geq 0,$$

where each linear weight *w* represents the importance of each kernel *K*, which is an expression function for cell *i* and cell *j*. In addition, SIMLR applies the following optimization framework to compute cell-to-cell similarity *S*:

$$\begin{array}{c} \text{minimize}\\ S,L,W \end{array}-\sum_{i,j,l}w_{l}\,K\,l\,\left(c_{i},c_{j}\right)\,S_{i\,j}+\beta \,{||S||}_{F}^{2}+\gamma \cdot t\,r\,\left(L^{T}\,\left(I_{N}-S\right)\,L\right)$$

$$+\rho \,\sum_{l}w_{l}\,l\,o\,g\,w_{l}$$

subject to

$$L^{T}\,L=I_{C}\,\sum_{j}w_{l}=1,w_{l}\geq 0,\sum_{j}S_{i\,j}=1\,\text{and}\,S_{i\,j}=0$$

where *I_N* and *I_C* are *N* × *N* and *C* × *C* identification matrices, respectively, and β and γ are non-negative tuning parameters; *L* denotes an auxiliary low-dimensional matrix enforcing the low rank constraint on *S*, *tr*(.) denotes the matrix trace, and |*S*|_*F*_ represents the Frobenius norm of *S*. The optimization problem has three variables: the similarity matrix *S*, the weight vector *w*, and an *N* × *C* rank-enforcing matrix *L*. SIMLR solves the optimization problem through updating each variable and fixing the other two variables.

Single-cell interpretation *via* multikernel learning used the stochastic neighbor embedding (SNE) method (Maaten and Hinton, 2008) to dimension reduction based on the cell-to-cell similarity *S* learned from the above optimization model. However, the objective function of SIMLR involves large-scale matrix multiplication, which leads to a large amount of calculation; thus, it is difficult to extend to high-dimensional datasets.

### Simulated scRNA-seq Datasets

To investigate the sensitivity of some characteristics of scRNA-seq datasets including cell type number, the number of cells and genes, outliers, and dropout event, we generated simulated datasets using the *Splatter* R package (Zappia et al., 2017). Function *splatSimulate()* is used to generate simulations, and *setParams()* is used to set specific parameters. First, we initialized the number of cell types as 5, the cell number as 2,000, the gene numbers as 5,000, and the probability of expression outlier as 0.05. When generating the simulated scRNA-seq data, we updated each parameter and fixed other parameters. Specifically, we generated the simulated data with variable numbers of cell types (5, 7, 9, 11, 13), cells (100, 500, 1,000, 2,000, 5,000, 10,000, 20,000, 30,000, 40,000, 50,000), genes (10,000, 20,000, 30,000, 40,000, 50,000), and probabilities of expression outliers (0.1, 0.2, 0.3, 0.4, 0.5). In addition, considering the impact of dropout, we also simulated datasets with five different levels of dropout (dropout.mid = −1, 0, 1, 2, 3, the larger the parameter, the more the points will be marked as 0); other parameters are set as default. Here, the probability of zero value in the data is 41, 53, 62, 71, and 80%, respectively. The detailed parameters are provided in Supplementary Table 1. In total, we created 30 simulated scRNA-seq datasets. The raw expression count matrices of these datasets are generated and normalized to suit for each investigated method.

### Real scRNA-seq Datasets

This study analyzed five real scRNA-seq datasets, all of which were downloaded from the publicly available EMBL or GEO databases (Supplementary Table 2). They are derived from different species and organs, covering a variety of cell types and data dimensions. Cell types of every dataset provided in original experiments were used as a gold standard to evaluate dimension reduction methods. The descriptions of all the scRNA-seq datasets are as follows:

1. Deng dataset: isolated cells from F1 embryos from oocyte to blastocyst stages of mouse preimplantation development with six cell types were collected and sequenced by Smart-Seq2 (Deng et al., 2014).
2. Chu dataset: single undifferentiated H1 cells and definitive endoderm cells (DECs) from human embryonic stem cells sequenced by SMARTer (Chu et al., 2016).
3. Kolodziejczyk dataset: mouse embryonic stem cells from different culture conditions with three cell types (Kolodziejczyk et al., 2015). Each library was sequenced by SMARTer.
4. Segerstolpe dataset: human pancreatic islet cells with 15 cell types obtained by Smart-Seq2 (Segerstolpe et al., 2016).

Additionally, we use PBMCs from a healthy human (PBMC68k dataset) (Zheng et al., 2017) generated by the 10X Genomics platform to assess the scalability of methods.

### Evaluation Metrics

To compare different dimension reduction methods, we performed the iterative k-means clustering on the low-dimensional representation of scRNA-seq data. Taking into account the randomness of k-means clustering when setting the initial cluster centroids, we performed k-means clustering 50 times to obtain a stable metric, and then set the cluster number k to the true cell type number. The evaluation metrics comparing the results to the true cell types are adjusted rand index (ARI), normalized mutual information (NMI), and Silhouette score.

Adjusted rand index (Santos and Embrechts, 2009) is a widely used metric which calculates the similarity between the two clustering results, which ranges from 0 to 1. A larger score means that two clusters are more consistent with each other. Conversely, when the clustering results are randomly generated, the score should be close to zero. Given two clustering X and Y,

$$\text{ARI}=\frac{\left(\begin{array}{c} n\\ 2 \end{array}\right)\,\left(a+d\right)-\left[\left(a+b\right)\,\left(a+c\right)+\left(c+d\right)\,\left(b+d\right)\right]}{\left(\begin{array}{c} n\\ 2 \end{array}\right)-\left[\left(a+b\right)\,\left(a+c\right)+\left(c+d\right)\,\left(b+d\right)\right]}$$

where *a* is the number of objects in a pair placed in the same group in X and in the same group in Y; *b* is the number of objects in a pair placed in the same group in X and in different groups in Y; *c* is the number of objects in a pair placed in the same group in Y and in different groups in X; and *d* is the number of objects in a pair placed in the different groups in Y and in different groups in X.

Normalized mutual information (Emmons et al., 2016) is used to estimate the concordance between the obtained clustering and the true labels of cells. NMI value is from 0 to 1. A higher NMI refers to higher consistency with the golden standard.

Specifically, given two clustering results X and Y on a dataset, NMI=*I*(*X*,*Y*/*max*{*H*(*U*),*H*(*V*)}, where

$$I\,\left(X,Y\right)=\sum_{x,y}p\,\left(x,y\right)\,\log\frac{p\,\left(x,y\right)}{p\,\left(x\right)\,p\,\left(y\right)}$$

$$U\,\left(X,Y\right)=\frac{2\cdot I\,\left(X,Y\right)}{H\,\left(X\right),H\,\left(Y\right)}$$

$$H\,\left(X\right)=\,\sum_{i=1}^{n}p\,\left(x_{i}\right)\,I\,\left(x_{i}\right)=\sum_{i=1}^{n}p\,\left(x_{i}\right)\,\log_{b}\frac{1}{p\,\left(x_{i}\right)}\,=-\sum_{i=1}^{n}p\,\left(x_{i}\right)\,\log_{b}p\,\left(x_{i}\right)$$

Silhouette coefficient (Aranganayagi and Thangavel, 2007) measures how well each cell lies with its own cluster, which indicates the separability of each individual cluster. The value of Silhouette coefficient s(*i*) is between −1 and 1; 1 means that the cell is far away from its neighboring clusters, whereas −1 means that the cell is far away from points of the same cluster.

$$s\,\left(i\right)=\frac{b\,\left(i\right)-a\,\left(i\right)}{max\{a\left(i\right),b\left(i\right)\}}$$

where *a*(*i*) is the average distance from cell *i* to other cells in the same cluster and *b*(*i*) is the average distance from cell *i* to all cells in other clusters. Average *s*(*i*) over all the cells indicates how separable each cell type in the low-dimensional representation, which we call the Silhouette score.

### Computing Cost

Computing cost of each method is estimated by monitoring the running time and peak memory usage. We analyzed the PBMC68k datasets from 10X Genomics. The raw count matrix was downsampled to 100, 500, 1,000, 2,000, 5,000, 10,000, 20,000, 30,000, 50,000, and 68,579 cells with 1,000 highly variable genes. All methods were run on the 10 downsampled datasets. We use the command *pidstat* from the sysstat tool to return the peak memory usage of the process in operation. When calculating the running time, we used the function *system.time()* in R. In this step, only the running time of the model is considered, and other processes such as data loading are excluded.

### Overall Performance Score

To rank methods, the overall scores of the methods were calculated through aggregating accuracy, stability, and computing cost (Zhang et al., 2020). After k-means clustering, we used the known cell populations to calculate the ARI, NMI, and Silhouette scores for simulated data and real data, respectively. For accuracy, scaled mean ARI, scaled NMI, and scaled Silhouette scores obtained from real data were aggregated to the accuracy score. For stability, aggregated scaled scores across different simulation datasets were denoted as the stability score of each method. For the computing cost, we first scale the running time and memory usage to get a value ranging from 0 to 1. Then, we averaged scaled running time and memory usage to obtain the computing cost. Finally, we integrated the accuracy, stability, and computing cost with a ratio of 40:40:20 into the overall performance score of each method.

## Results

We benchmarked a total of 10 methods on 30 simulated and five real datasets. We normalized scRNA-seq data based on the corresponding method, and then performed dimensionality reduction to obtain 2D latent space. k-Means clustering method was used to perform cluster analysis. Finally, the methods were compared using accuracy, stability, computing cost, and sensitivity to hyperparameters (Figure 1).

### Evaluation of Stability

We used 30 simulated datasets to assess the stability of the 10 dimensionality reduction methods with respect to the number of cell type, cells and genes, outliers, and dropout event.

**FIGURE 2:**
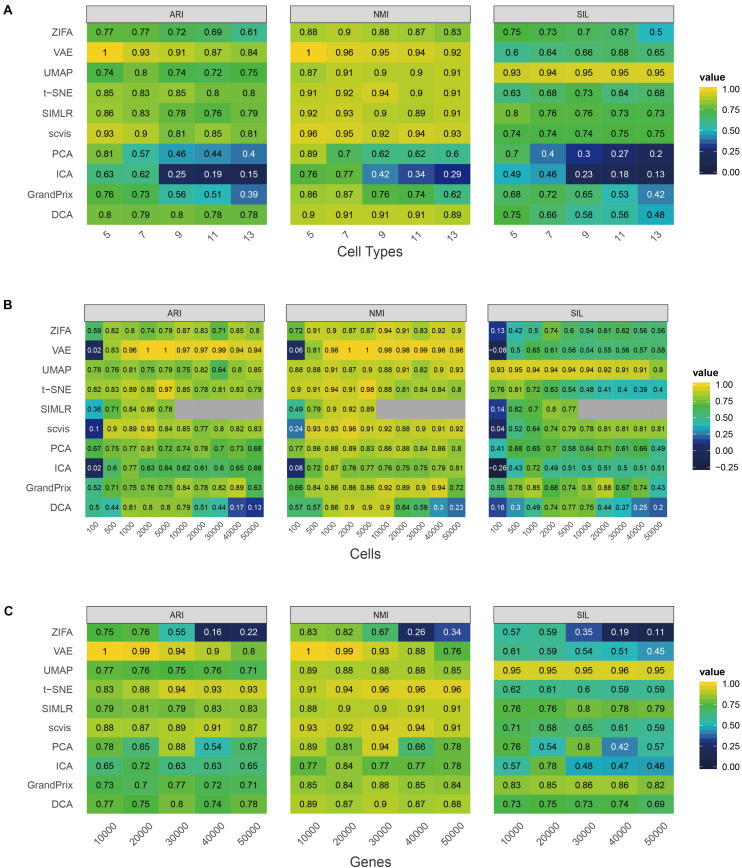
Evaluation stability of the 10 dimensionality reduction methods on simulated scRNA-seq data with respect to the number of cell type **(A)**, cell number **(B)**, or gene number **(C)**. The performance is measured by ARI, NMI, and Silhouette score (SIL). Gray indicates that the SIMLR cannot run on data with more than 10,000 cells.

First, we investigated the effect of cell type numbers to the approaches. We fixed the cell number (*n* = 2,000), gene number (*n* = 5,000), and probability of outliers (*p* = 0.05), and then changed the cell type number from 5 to 13 stepped by 2. As the number of cell types increased, the performance of PCA, ICA, and GrandPrix descended faster (Figure 2A). While the performance of ZIFA, VAE, SIMLR, scvis, and DCA decreased slightly, UMAP and t-SNE fluctuated. Generally, ZIFA, VAE, SIMLR, scvis, DCA, UMAP, and t-SNE have better stability with respect to cell type number than PCA, ICA, and GrandPrix, since their standard deviation is relatively small.

Second, we changed the cell number from 100 to 50,000 and fixed other factors. It was found that too many or too few cells are not conducive to the construction of low-dimensional space of single-cell RNA-seq data. All the methods’ performance fluctuated greatly except for PCA and UMAP. PCA and UMAP have strong adaptability to cell number change based on standard deviation (Figure 2B). All of the methods obtained the best performance between 1,000 and 10,000 cells. It is worth noting that SIMLR has a high computational complexity as it involves large matrix operations which could not perform dimensionality reduction on data with a cell count greater than or equal to 10,000. Additionally, all the methods except PCA and ZIFA have good stability with respect to gene number (Figure 2C).

**FIGURE 3:**
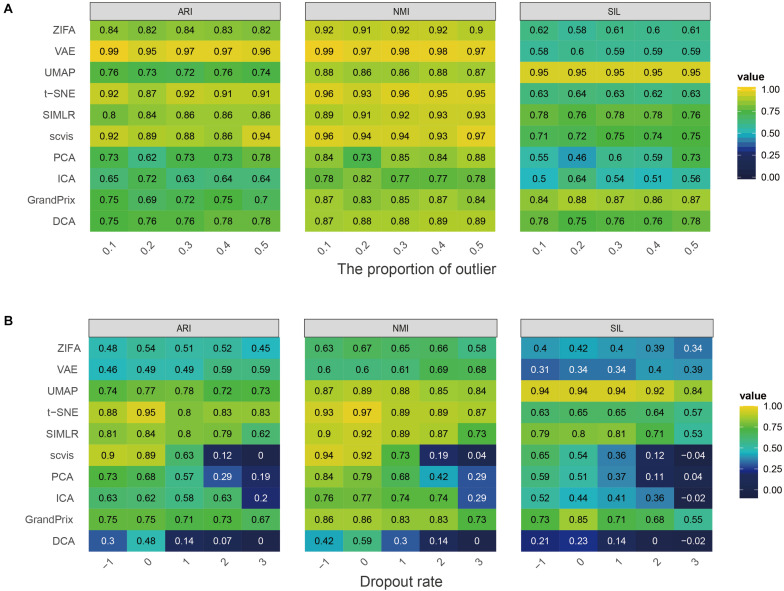
Evaluation stability of the 10 dimensionality reduction methods on simulated scRNA-seq data with respect to the proportion of outlier **(A)** or dropout rate **(B)**. The performance is measured by ARI, NMI, and SIL.

To investigate the effect of the complex cell mixtures to methods, we simulated expression outliers; it was found that the performance of all the methods is stable to expression outliers (Figure 3A). Finally, we randomly dropped expressed genes in each cell to investigate the ability of methods to deal with datasets with various library sizes. Generally, ZIFA, VAE, UMAP, t-SNE, SIMLR, and GrandPrix showed a stable performance, whereas the performance of scvis, PCA, ICA, and DCA decreased remarkably with the increase in the dropout ratio (Figure 3B).

**FIGURE 4:**
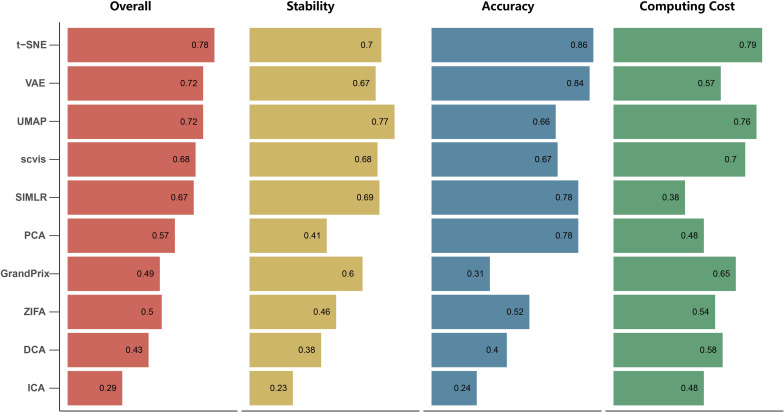
The overall performance of the 10 dimensionality reduction algorithms. The methods are sorted by overall performance score, which is a weighted integration of accuracy, stability, and computing cost. The accuracy and stability are the average value of scaled ARI, scaled NMI, and scaled SIL in real data and simulated data, respectively. Running time and memory are scaled to a value in [0,1] before averaged as computing cost.

We found that the stability of each method is different with respect to the number of cell types, cells and genes, outliers, and dropout rate. To evaluate the overall stability of each method, we aggregated all the metrics across simulation datasets to obtain the overall stability score (see section “Materials and Methods”). In summary, the overall stability scores showed that the performance of UMAP has shown more stability than the other methods. Conversely, ICA has poor stability (Figure 4). It is worth mentioning that the Silhouette score of UMAP is significantly higher than the other methods in all simulation tests, indicating that it better separated distinct cell types.

### Evaluation of Accuracy

**FIGURE 5:**
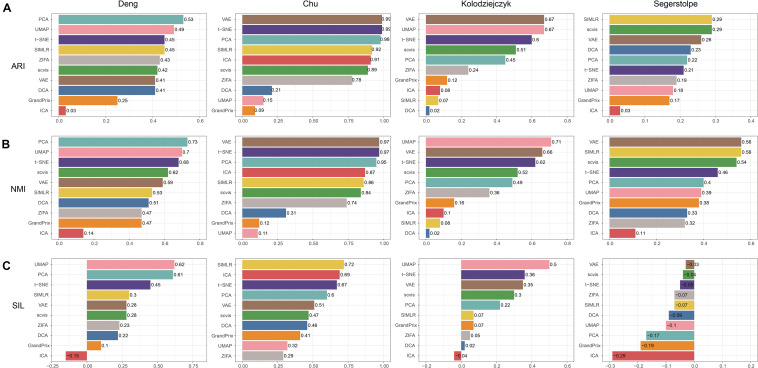
Evaluation accuracy of the 10 dimensionality reduction methods on real scRNA-seq data measured by **(A)** ARI, **(B)** NMI, and **(C)** SIL.

We applied the 10 dimensionality reduction methods to the four real data and performed k-means cluster analysis based on the low-dimensional representation and calculated the evaluation metrics. No single method dominated on all of these datasets, indicating that there is no “one-size-fits-all” method that works well on every dataset. Regarding the ARI and NMI measures, PCA and t-SNE were ranked in the top five performers on all the four datasets (Figures 5A,B). VAE was ranked in the top five performers on the three datasets. Consistent with the simulation dataset, UMAP can separate each individual cluster very well based on the Silhouette score, compared with other methods (Figure 5C). In addition, the dataset of Segerstolpe et al. has the lowest evaluation metrics compared with the other three datasets, indicating that the dimensionality reduction method should be improved for the heterogeneous dataset with more cell types. We also visualized the low-dimensional reductions of all the methods on the four datasets (Supplementary Figures 1–4). The ability to separate different cell types of each method is consistent with the above metrics. Aggregating all the three metrics across datasets, t-SNE has the best accuracy, followed by VAE (Figure 4).

### Sensitivity of Methods to Hyperparameters

**FIGURE 6:**
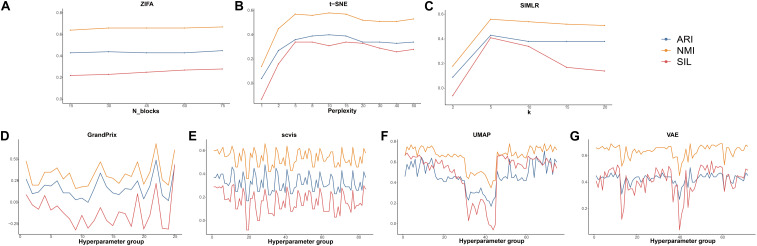
The effect of hyperparameters to the performance of dimensionality reduction methods. **(A)** ZIFA. **(B)** t-SNE. **(C)** SIMLR. **(D)** Grandprix. **(E)** Scvis. **(F)** UMAP. **(G)** VAE.

The hyperparameters play a crucial part of the dimension reduction algorithm, especially the deep machine learning model. Therefore, we examined the effect of the hyperparameter settings on the dimensionality reduction in order to guide the user in making a reasonable choice. Among all the 10 algorithms discussed, there are seven methods whose developers have added parameter settings. PCA and ICA are based on linear transformations, so do not require hyperparameter adjustment. In addition, DCA implements an automatic search that could identify a set of hyperparameters in minimizing errors. To decrease time consumption, we used the datasets of Deng to investigate the effect of the hyperparameters to the performance of these seven methods. Detailed evaluation parameters are shown in Supplementary Table 3. Using grid search strategy, we found that ZIFA is insensitive to their respective hyperparameters, and the evaluation metrics have little change in different settings (Figure 6A). The evaluation metrics of t-SNE and SIMLR increased when their hyperparameters increased from 2 to 5, after that ARI and NMI tend to be stable. Silhouette scores are largely reduced when the hyperparameters are larger than 20 (Figures 6B,C). For those methods with multiple adjustable hyperparameters including GrandPrix, scvis, UMAP, and VAE, we noticed a dramatic change in the results when choosing different hyperparameter settings (Figures 6D–G). Therefore, we recommend that users consider the impact of hyperparameter settings before using these four methods.

### Data Preprocessing of All Methods

For the arithmetic design adapting to different algorithms, we performed the corresponding normalization process for one raw single-cell RNA-seq data based on the description of the algorithm. First, PCA, ICA, t-SNE, UMAP, ZIFA, and SIMLR used the original count matrix of scRNA-seq data as the input. For DCA and GrandPrix, the input is a feature matrix with all the cells and 1,000 highly variable genes. Scvis used PCA as a preprocessing for noise reduction to project the cells into a 100-dimensional space.

### The Outputs of All Methods

For some methods, in addition to the low-dimensional representation of the data, other useful information is also provided. Specifically, scvis, DCA, and VAE were developed based on deep learning; thus, a trained model is saved in the corresponding output folder, containing the loss parameters and validation for models. Furthermore, being used as a process of noise reduction, DCA provides an output file which represents the mean parameter of the ZINB distribution which has the same dimensions as the input file. Detailed workflows and explanations are available in the original publications.

### Computing Cost Overview

**FIGURE 7:**
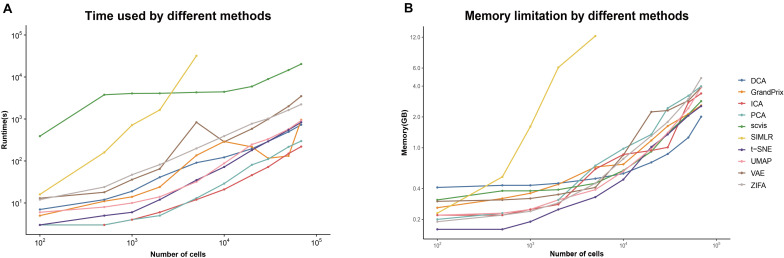
Evaluation computing cost for each method on metrics of **(A)** running time and **(B)** memory limitation. The analyses were run on computing equipment with Inter i7 4790@3.60 GHz CPU and 16G running memory.

The current scRNA-seq analysis methods are expected to cope with hundreds of thousands of cells as the number of cells profiling by the current protocols increases. We estimated the computational efficiency of each method using running time and memory usage. We generated ten datasets containing different number of cells through downsampling the PBMC68k data. Overall, the running time and memory usage of all methods are positively correlated with the cell number. Most methods except SIMLR and scvis can be completed in 30 min even using all the cells of PBMC68k dataset (Figure 7A). Most methods except SIMLR and ZIFA can complete all the processes within 4 GB (Figure 7B). We noted that SIMLR is difficult to be performed on the dataset with more than 10,000 cells due to its unique multikernel matrix operation. In general, ICA took the shortest time (3.7 min) and t-SNE had the lowest memory requirements (2.5 GB) when the number of cells is 68k. Overall, t-SNE has the best computing cost (Figure 4).

### Overall Performance

By integrating three metrics from measurement of accuracy, stability, and computing cost, we obtained the overall performance score for each method (Figure 4). We found that t-SNE achieved the best overall performance score with the highest accuracy and computing cost. Meanwhile, UMAP exhibited the highest stability, as well as moderate accuracy and the second highest computing cost. However, the performance score of these methods is different across evaluation criteria. For example, SIMLR and PCA performed better than UMAP based on accuracy, while SIMLR showed weaker computing cost and PCA showed weaker stability.

## Discussion

Since 2015, the emergence of 10X Genomics, Drop-seq, Micro-well, and Split-seq technologies has completely reduced the cost of single-cell sequencing. This technology has been widely used in basic scientific and clinical research. An important application of single-cell sequencing is to identify and characterize new cell types and cell states. In this process, the key question is how to measure the similarity of the expression profiles of a set of cells, whereas, such similarity analysis can be improved after reducing dimensionality, which can help in noise reduction.

Here, we performed a comprehensive evaluation of 10 dimensionality reduction methods using simulation and real dataset to examine the stability, accuracy, computing cost, and sensitivity to hyperparameters. Taken together, we observed that the summarized performance of t-SNE outperformed the performance of other methods. UMAP has the highest stability and can separate distinct cell types very well. Although, both methods are not specifically designed for single-cell expression data. However, the performance of most methods decreased as cell number and dropout rate increased. Therefore, new algorithms will likely be needed to effectively deal with dropout rate and millions of cells. In addition, the dataset from Segerstolpe et al. containing the lower evaluation metrics showed that the dimensionality reduction method should be improved for the heterogeneous dataset with more cell types. We suggested that users adjust the hyperparameters when using these non-linear and neural network methods. Finally, basic linear methods such as PCA and ICA have shown to be most time saving but perform worse in highly heterogeneous data.

To conclude, we provide a new procedure for comparing single-cell dimensionality reduction methods. We hope that this will be useful in providing and giving method users and algorithm developers an exhaustive evaluation of different data and appropriate recommendation guidelines. At the same time, new dimensionality reduction methods are being developed which will become more robust and standardized. These developments will deepen further exploration and comprehensive understanding of single-cell RNA-seq applications.

## Data Availability Statement

The original contributions presented in the study are included in the article/Supplementary Material. Further inquiries can be directed to the corresponding author/s.

## Author Contributions

RX performed data analysis, data visualization, and manuscript writing. WW, LY, and SW participated in the discussion. CX and XC supervised the project and revised the manuscript. All authors contributed to the article and approved the submitted version.

## Conflict of Interest

The authors declare that the research was conducted in the absence of any commercial or financial relationships that could be construed as a potential conflict of interest.
