# Supplementary material for: A Comparison for Dimensionality Reduction Methods of Single-Cell RNA-seq Data
Source: Front Genet. 2021 Mar 23;12:646936. doi: 10.3389/fgene.2021.646936 (PMC8021860; doi:10.3389/fgene.2021.646936)
Supplement: Supplementary file 1 [file Data_Sheet_1.docx]

Supplementary Material

1. **Supplementary Figures and Tables**

## Supplementary Figures


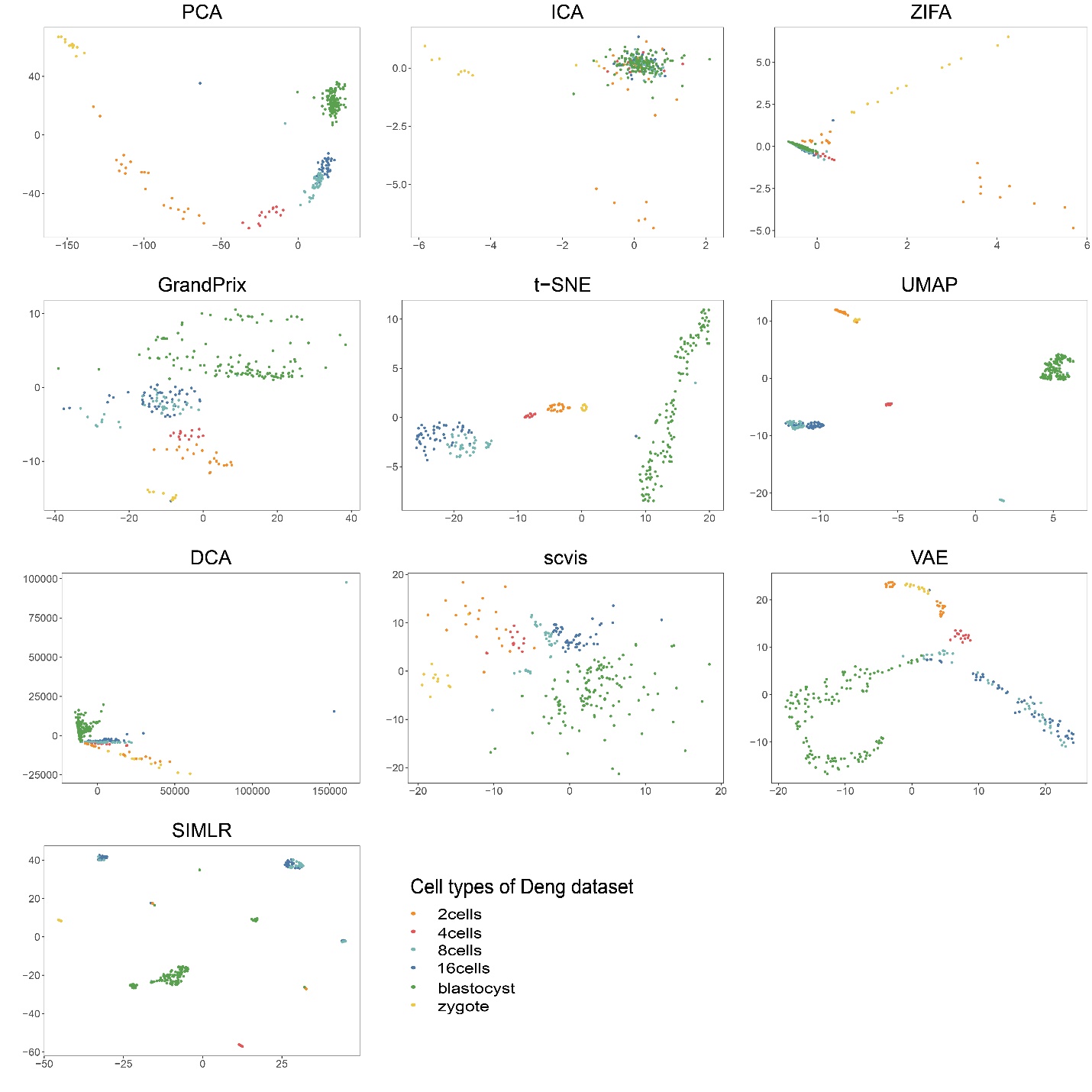


**Supplementary Figure 1. Comparison of 2D visualization on the Deng dataset.** Each point denotes a cell, which was isolated from the different developmental stages of in vivo F1 embryos including two-cell stage (2cells), four-cell stage (4cells), eight-cell stage (8cells), blastocyst and zygote stages. The true label information was provided in the form of different colors.

**
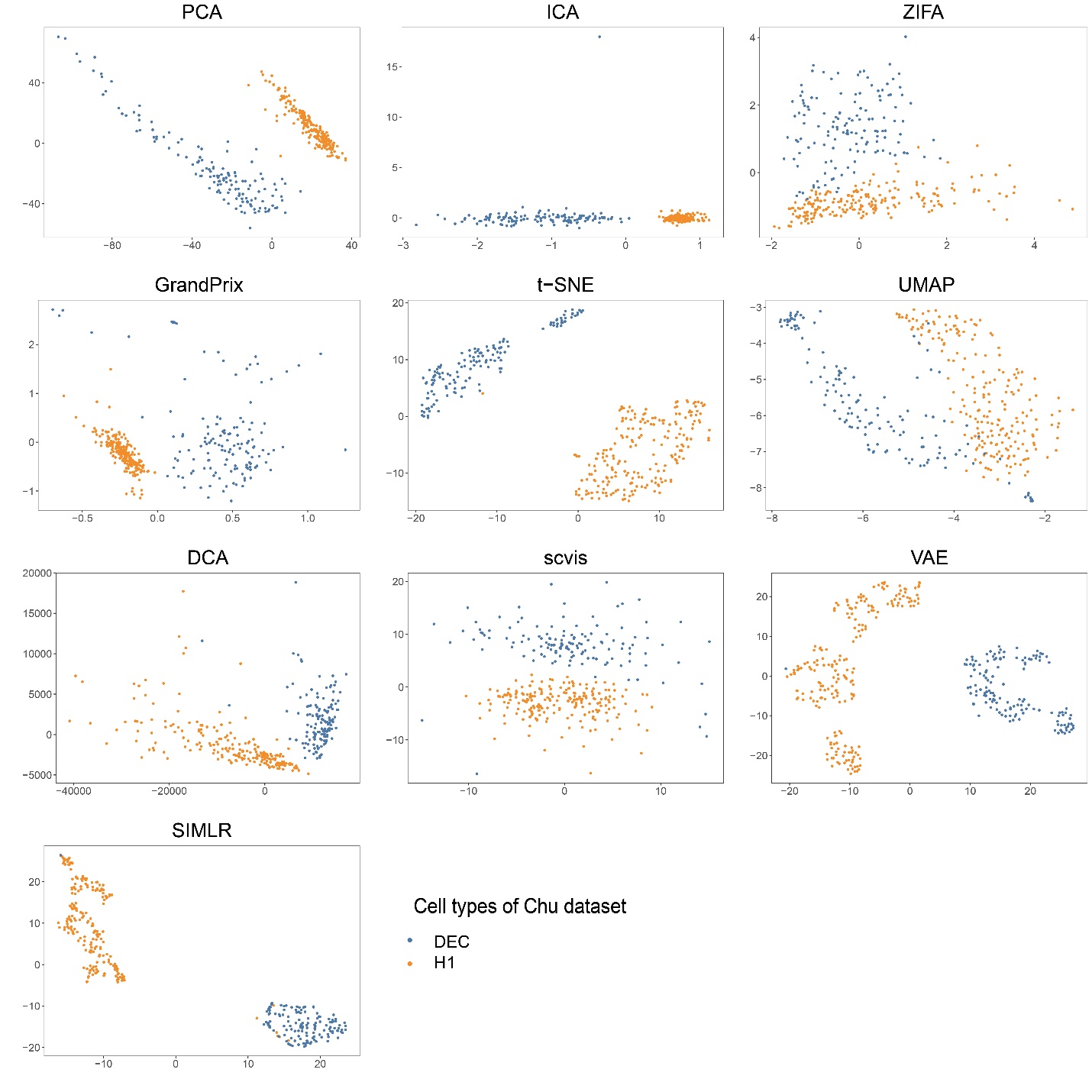
**

**Supplementary Figure 2. Comparison of 2D visualization on the Chu dataset.** Points denote cells, which are single undifferentiated H1 cells and definitive endoderm cells (DECs) from human embryonic stem cells. The true label information was provided in the form of different colors.

**
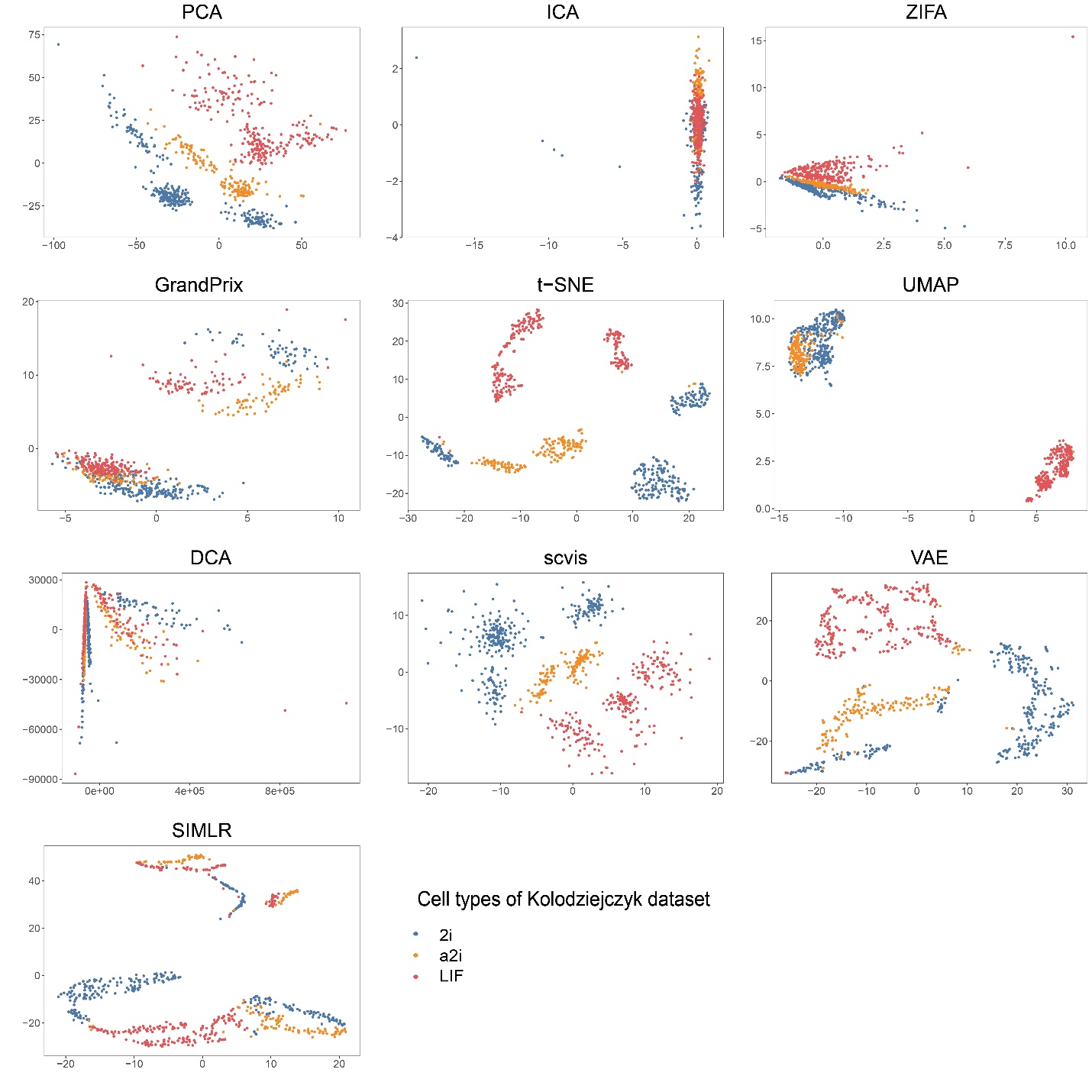
**

**Supplementary Figure 3. Comparison of 2D visualization on the Kolodziejczyk dataset.** Points denote cells, which were derived from mESC cultured under three different conditions: serum + leukemia inhibitory factor (LIF), 2i, and the alternative ground state a2i. The true label information was provided in the form of different colors.

**
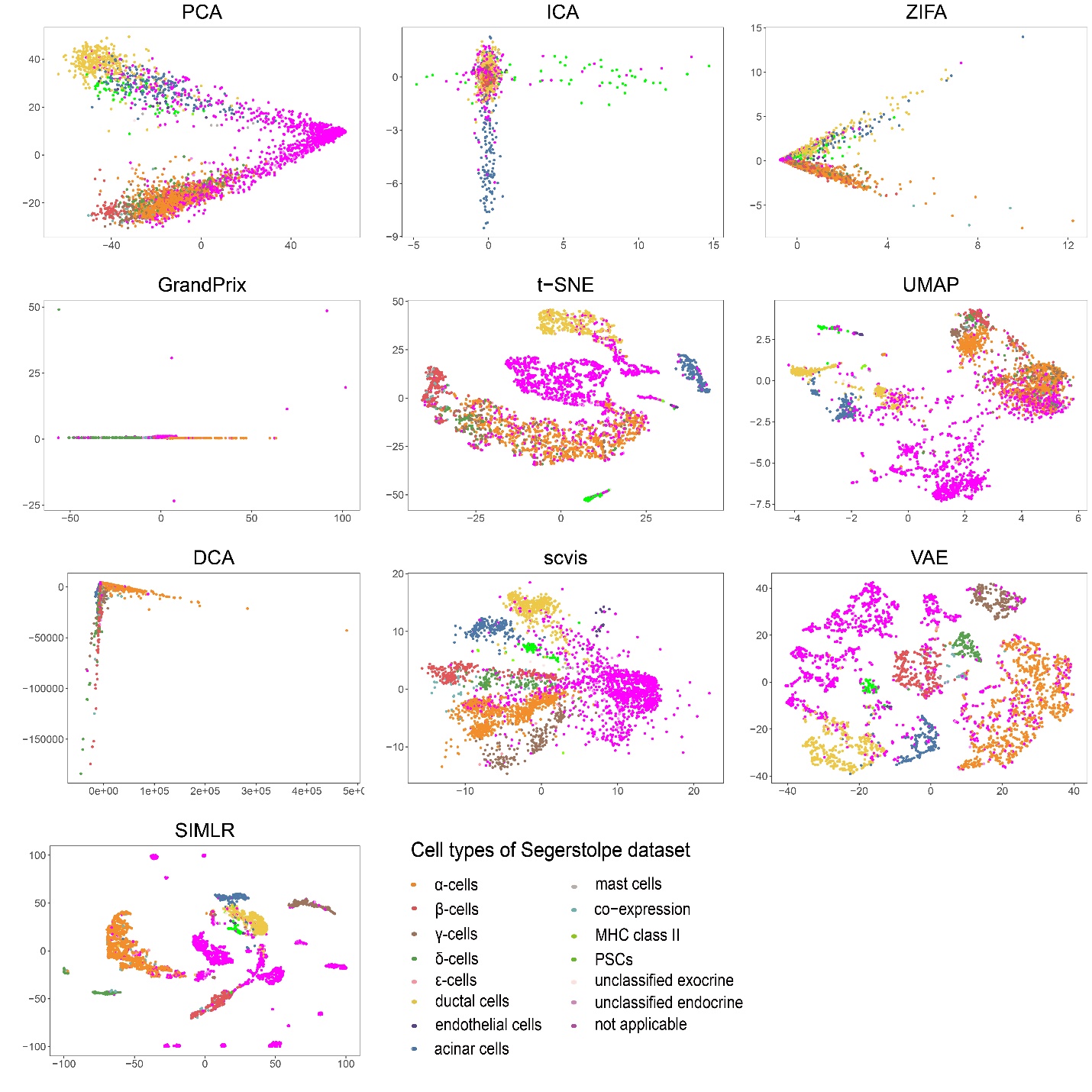
**

**Supplementary Figure 4. Comparison of 2D visualization on the Segerstolpe dataset.** Points denote cells, which are from Human pancreas. The true label information was provided in the form of different colors.

## Supplementary Tables

**Supplementary Table 1. Summary of simulation scRNA-seq datasets.**

| Simulation Dataset | Cell Type | Cell | Gene | Probability of Outliers | Dropout.mid  (dropout.type=  ” experiment”) |
| --- | --- | --- | --- | --- | --- |
| 1 | 5 | 2000 | 5000 | 0.05 | / |
| 2 | 7 | 2000 | 5000 | 0.05 | / |
| 3 | 9 | 2000 | 5000 | 0.05 | / |
| 4 | 11 | 2000 | 5000 | 0.05 | / |
| 5 | 13 | 2000 | 5000 | 0.05 | / |
| 6 | 5 | 100 | 5000 | 0.05 | / |
| 7 | 5 | 500 | 5000 | 0.05 | / |
| 8 | 5 | 1000 | 5000 | 0.05 | / |
| 9 | 5 | 2000 | 5000 | 0.05 | / |
| 10 | 5 | 5000 | 5000 | 0.05 | / |
| 11 | 5 | 10000 | 5000 | 0.05 | / |
| 12 | 5 | 20000 | 5000 | 0.05 | / |
| 13 | 5 | 30000 | 5000 | 0.05 | / |
| 14 | 5 | 40000 | 5000 | 0.05 | / |
| 15 | 5 | 50000 | 5000 | 0.05 | / |
| 16 | 5 | 2000 | 10000 | 0.05 | / |
| 17 | 5 | 2000 | 20000 | 0.05 | / |
| 18 | 5 | 2000 | 30000 | 0.05 | / |
| 19 | 5 | 2000 | 40000 | 0.05 | / |
| 20 | 5 | 2000 | 50000 | 0.05 | / |
| 21 | 5 | 2000 | 5000 | 0.1 | / |
| 22 | 5 | 2000 | 5000 | 0.2 | / |
| 23 | 5 | 2000 | 5000 | 0.3 | / |
| 24 | 5 | 2000 | 5000 | 0.4 | / |
| 25  26  27  28  29  30 | 5  5  5  5  5  5 | 2000  2000  2000  2000  2000  2000 | 5000  5000  5000  5000  5000  5000 | 0.5  0.05  0.05  0.05  0.05  0.05 | /  -1  0  1  2  3 |

**Supplementary Table 2. Summary of real scRNA-seq datasets.**

| Dataset | Data Source | Protocol | Date | Cell | Gene | Cell Type | Specie | Cell Resource |
| --- | --- | --- | --- | --- | --- | --- | --- | --- |
| Deng | GEO（GSE45719） | [Smart-Seq2](http://dx.doi.org/10.1038/nprot.2014.006) | 2014 | 268 | 22431 | 6 | Mouse | Embryonic devel cells |
| Chu | GEO（GSE75748） | SMARTer | 2016 | 350 | 17507 | 2 | Human | Embryonic stem cells |
| Kolodziejczyk | EMBL-EBI  （E-MTAB-2600） | SMARTer | 2017 | 704 | 38653 | 3 | Mouse | Embryonic stem cells |
| Segerstolpe | EMBL-EBI  （E-MTAB-5061） | [Smart-Seq2](http://dx.doi.org/10.1038/nprot.2014.006) | 2016 | 3514 | 25525 | 15 | Human | Pancreas |
| PBMC68k | / | 10x Genomics | 2017 | 68579 | 32738 | 8 | Human | Peripheral blood mononuclear cells |

**Supplementary Table 3. Adjustment strategy for hyperparametric test.** The setting of hyperparameters is based on all different combinations of the parameters.

| Method | Evaluation combinations of hyperparameters |
| --- | --- |
| GrandPrix | ls = {0.01, 0.1, 0.5, 1, 2}  var = {0.01, 0.1, 0.5, 1, 2}  **default:** ls = 1, var = 1 |
| Scvis | layer_number = {10-1, 10-3, 10-5, 10-7, 10-10, 7-10, 5-10, 3-10, 1-10}  unit_number = {8, 16, 32, 64, 128}  perplexity = {0, 0.5, 1, 1.5, 2, 10}  **default:** layer_number = 3-5, unit_number = [128, 64, 32],[32, 32, 32, 64, 128], perplexity = 10 |
| SIMLR | k = {2, 5, 10, 15, 20}  **default:** k = 10 |
| t-SNE | perplexity = {1, 2, 5, 8, 10, 15, 20, 30, 40, 50}  **default:** perplexity = 30 |
| UMAP | n_neighbor = {2, 5, 10, 20, 100}  distance = {0, 0.1, 0.25, 0.5, 1}  metrics = { ‘correlation’, ‘euclidean’, ‘minkowski’}  **default:** n_neighbor = 15, distance = 0.1, metrics = ‘euclidean’ |
| VAE | batch_size = {50, 100, 200}  epoch = {25, 50, 100, 200}  first_layer = {100, 250, 500}  d={2, 3}  **default:** batch_size = 50, epoch = 200, first_layer = 2 |
| ZIFA | N_blocks = {15, 30, 45, 60, 75}  **default:** N_blocks = ngene/5 |
